# A New Approach for Spontaneous Silver Ions Immobilization onto Casein

**DOI:** 10.3390/ijms20163864

**Published:** 2019-08-08

**Authors:** Oleksandra Pryshchepa, Gulyaim N. Sagandykova, Paweł Pomastowski, Viorica Railean-Plugaru, Anna Król, Agnieszka Rogowska, Agnieszka Rodzik, Myroslav Sprynskyy, Bogusław Buszewski

**Affiliations:** 1 Department of Environmental Chemistry and Bioanalytics, Faculty of Chemistry, Nicolaus Copernicus University in Torun, 87-100 Torun, Poland; 2 Interdisciplinary Center for Modern Technologies, Nicolaus Copernicus University in Torun, 87-100 Torun, Poland

**Keywords:** binding kinetics, casein, sorption, kinetics, isotherms, metalloproteins, silver nanoparticles, casein-silver nanocomplex

## Abstract

The work presents the kinetic and isotherm studies of silver binding on casein, which was carried out using batch sorption technique. Moreover, the influence of light irradiation on the process was shown. In order to investigate the mechanism of metal ions sorption by casein the zero, pseudo-first order kinetics and Weber-Morris intra-particle diffusion as well as Langmuir and Freundlich isotherm models were used. Furthermore, to specify more precisely, the possible binding mechanism, the spectroscopic (FT-IR—Fourier Transform Infrared Spectroscopy, Raman), spectrometric (MALDI-TOF MS—Matrix-Assisted Laser Desorption/Ionization Time Of Flight Mass Spectrometry), microscopic (SEM—Scanning Electron Microscope, TEM/EDX—Transmission Electron Microscopy/Energy Dispersive X-ray detector) and thermal (TGA—Thermogravimetric Analysis, DTG—Derivative Thermogravimetry) analysis were performed. Kinetic study indicates that silver binding onto casein is a heterogeneous process with two main stages: initial rapid stage related to surface adsorption onto casein with immediate creation of silver nanoparticles and slower second stage of intraglobular diffusion with silver binding in chelated form (metalloproteins) or ion-exchange form. Spectroscopic techniques confirmed the binding process and MALDI-TOF MS analysis show the dominant contribution of the α-casein in the process. Moreover, the treatment of silver-casein complex by artificial physiological fluids was performed.

## 1. Introduction

Milk is a valuable source of bioactive ingredients with a positive effect on human and other mammalian health. Moreover, it is high-quality and the only nourishment in the diet of mammals from the first days of life [1]. Milk mainly consists of casein, which makes up about 80% of the total milk proteins, and the other fraction is serum or whey proteins [2]. Casein consists of four gene products: α_s1_-, α_s2_-, β-, and κ-casein, which differ in structure and degree of posttranslational modification. Casein is resistant to high temperatures, but susceptible to digestive enzymes. Casein is a protein, which elemental composition contain not only carbon (53%), hydrogen (7%), oxygen (22%), nitrogen (15.60%), sulphur (0.78%), but also phosphorus (0.86%). In milk, caseins are the part of phosphoprotein structures, which appears in form of large colloidal aggregates called casein micelles [3]. Such composition and structure ensures the main milk function, namely the effective delivery of calcium, phosphate, and protein from the mammary gland to the offspring [4].

Casein micelles are the spherical particles in the range from 50 to 600 nm in diameter and with an average diameter of about 200 nm [5]. Casein micelle consists of protein in about 93%, and the rest are inorganic compounds such as salts of calcium, magnesium, sodium, and potassium with phosphate and citrate acid residues, that are collectively known as colloidal calcium phosphate [3]. One of the most important casein functions is to dissolve calcium and phosphate during lactation in the glands. Nevertheless, the casein micelle size does not depend on the calcium and phosphate content, but is determined by the composition of the milk: casein and whey protein content, the composition of different types of caseins and therefore from genetic traits of species, animal nutrition or season [5]. Calcium is essential for micelle formation; which properties change with changes in calcium concentration. After adding a small amount of calcium, soluble caseins initially increase the micellar mass without increasing its size, provided that casein micelles can incorporate caseins insensitive to calcium. Further addition of calcium leads to increased micellar radius as long as critical concentration is reached, then additional calcium might result in loss of aggregative stability and precipitation of micelles [4].

The dispersion stability and other colloidal properties of casein micelles depend from their structure. Despite the large amount of research on casein micelles, their exact structure and stability are still not fully known and are constantly discussed, which is emphasized in the literature. Several models of casein structure have been proposed. The oldest model of casein micelle is the submicelle model described by Slattery and Evard with Schmidt’s [6]. Main hypothesis in submicelle model based on an assumption that casein micelles are made from assembled small subunits (submicelles), which are formed with 15–20 protein molecules through hydrophobic interactions. The micelle formation implies existence of k-casein-rich submicelles located on the surface and k-casein-deficient submicelles inside, and all the system is stabilized by colloidal calcium phosphate [6]. Another model is the Holt’s nanocluster model, where casein micelle is considered as a homogeneous casein matrix in which small colloidal calcium phosphate nanoclusters are dispersed. The nanoclusters form the central element of the casein micelle structure. Phosphoseryl clusters of the calcium-sensitive caseins are the interaction sites that can bind to calcium phosphate nanoclusters surface. Considering that α_s1_- and α_s2_-caseins have more than two such clusters they are able to form a 3-dimensional cross-linked network structure. Moreover, sticking out protein tails can bind to other proteins through weak interactions, i.e., hydrophobic interactions, hydrogen bonding, ion bonding and weak electrostatic interactions, to obtain a homogeneous protein matrix [6,7]. The first and second models are critically evaluated, as they turn out to be an extension of the third model—the dual-binding model proposed by Horne. In the dual-binding model, calcium phosphate through binding to phosphoseryl clusters not only forms cross-links, but also reduce proteins charge, which makes the attractive interactions between hydrophobic regions a dominant force [8]. This model confirms the rheomorphic concept of the second model and emphasizes the amphiphilic character of casein, in which caseins behave like block copolymers formed by charged and hydrophobic segments [9].

Metal ions are an extremely important element of many biological systems, and play an essential role in the functioning of many proteins. Nowadays, an important issue that attracts increasing attention in the field of chemistry is an interaction of proteins with metals [10]. Scientists interest put on metal-protein complexes both as an essential structures of the organisms and as a result of interactions with metal ions that are present in the environment [10,11]. Silver is an example of metal that extensively utilized in the research. Silver is known as antimicrobial agent for a long time, therefore its interaction with biopolymers is being studied [11]. According to Pearson’s Hard Soft (Lewis) Acid Base (HSAB) principle Ag^+^ ions belong to “soft” cations, which prefer binding to soft ligands. It suggests that silver will preferably bind to methionine or cysteine side chains, as sulfur-containing groups are the softest ligands [12]. Additionally, Ag^+^ ions show high affinity to phosphate, carboxyl, amino and imidazole functional groups. Protein structure also has a big influence on mechanism of its interactions with metal [13]. However, the exact processes that take place are still uncertain.

Despite Ag^+^ ions positive effect in the antimicrobial applications, it also shows cytotoxic properties. Silver preparations as a biocolloid (metalloproteins or nanocomplexes) may deal with metal ions cytotoxic properties [13]. Caseins are the proteins that can be used for such purposes. They are abundant in milk, which is a relatively low-cost raw material, so it can ensure high-scale and low-cost production. Moreover, there are reports about bacteriagenic silver nanoparticles, which were synthesized with usage of bacterial culture supernatant [14,15]. There is an assumption that bacterial culture supernatant contains bacterial metabolites which are responsible for reduction of the silver and therefore for nanoparticles creation [16]. However, some culture media contain casein or its hydrolysate, which could have an influence on the process of silver nanoparticles formation. In addition, the casein-silver (Ag-CN) complexes toxicity should be studied further, considering their speciation, and subsequently, potential toxic effects in the body.

The characterization of Ag^+^ ions interaction with casein is crucial for the process development of possible synthesis on an industrial scale of silver-protein nanocomplex—the potential efficient and cheap antiseptic agent. Casein is a component of some systems that can be utilized for synthesis of silver nanocomplexes, so it is necessary to depict possible processes that may take place. However, casein has ultra-complex structure compared to regular globular proteins, which can affect its interactions with metals. Therefore, the main goal of this work was to study the specificity of silver bonding/immobilization onto casein in aqueous solutions (kinetic and isotherm processes), as well as the process of silver-casein nanocomplex formation under different experimental conditions (in dark and lightened environment). In order to establish the possible ways of Ag^+^ ions to casein binding spectroscopic (FT-IR, Raman), spectrometric (MALDI-TOF MS), microscopic (SEM, TEM/EDX) and thermal (TGA, DTG) analysis were performed. In addition, the stability of the complex in synthetic physiological fluids was studied to evaluate its safety in case of accidental human and animal oral exposure.

## 2. Results and Discussion

### 2.1. Kinetics and Isotherm Study

**Figure 1:**
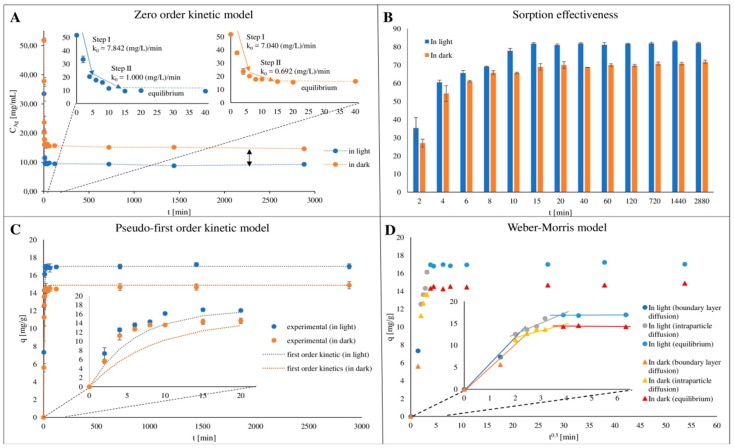
Kinetics of the silver ions onto casein sorption process in light and dark conditions. The kinetic steps of the Ag^+^ ions sorption by casein and values of the rate constants determined using zero order kinetic model (**A**); sorption effectiveness of the Ag^+^ ions by casein (**B**); experimental data and fitted pseudo first-order kinetics models of the Ag^+^ ions sorption by casein (**C**); and plot of intra-particle diffusion model of the Ag^+^ ions sorption onto casein (**D**).

In order to examine the mechanism of silver ions binding onto casein, the kinetic and isotherm approaches have been applied. Moreover, in order to determine the influence of light on the process, the kinetic study was performed in the presence of light and in the dark. Figure 1A presents the kinetic of the Ag^+^ ions sorption process, as a plot of Ag^+^ ions concentration changes in the solution, and Figure 1B shows the sorption effectiveness per unit time, depending on light conditions. The obtained results indicate that silver adsorption process in both conditions is not linear, and three separate steps can be identified. The first step is related to (i) rapid initial sorption, the second is connected with (ii) gradual sorption and the last one is (iii) sorption equilibrium. However, the presence of light has a significant influence on the effectiveness of studied process: the silver ions concentration decrease occurs more intensively. Such results may be caused by the reduction of silver ions under lightened conditions, but this difference is relatively low.

The maximum sorption effectiveness and capacity of casein have been achieved under light conditions (82.04 ± 0.50% and 17.01 ± 0.29 mg/g, respectively). In turn, these values obtained for dark conditions are 71.84 ± 0.94% and 14.88 ± 0.09 mg/g, respectively. Moreover, for both cases the first rapid stage of sorption occurs in the first 4 min of the process. In this step the sorption effectiveness was 65.58 ± 1.44% and 54.31 ± 4.33% and the casein sorption capacity was 12.54 ± 0.42 mg/g and 11.26 ± 0.97 mg/g for light and dark conditions, respectively. The sorption process in the second stage was much slower under both conditions and ends after 15 min of incubation. During this step the effectiveness of Ag^+^ ions sorption by casein increased up to 81.74 ± 0.61% and 68.99 ± 1.82% and the sorption capacity up to 16.95 ± 0.28 mg/g and 14.31 ± 0.55 mg/g for light and dark conditions, respectively. In both cases, the system reaches equilibrium 15 min after the beginning of the sorption process. The zero order kinetics model was applied to calculate the rate constants of Ag^+^ ions sorption kinetics for the linear segments of the first and second stages [13,17]. This model is suitable for describing separate sorption steps, which is characterized by a linear relationship. The velocity constant unit obtained using this model is a real physical parameter that characterizes the speed of the process. The rate constant values were summarized in Table 1. For the first step the rate constant values were calculated as 7.84 and 7.04 (mg/L)/min for light and dark conditions, respectively. For the second step, these constants were 1.00 and 0.69 (mg/L)/min, respectively. It can be observed that in both cases the speed of the first, initial, step is definitely higher than the speed of the second step.

In order to present the received experimental data more accurately, the pseudo first-order kinetics model was used. Figure 1C presents the matching of experimental data to pseudo first-order kinetics model and Table 1 summarize the calculated kinetics constants. Analysis of relative approximation error (A_approx._) values allow to conclude that the pseudo-first order kinetic model is more appropriate for description of the silver ions sorption process onto casein in the presence of light. The average values of relative approximation error were 13.29% and 26.58% for pseudo first-order kinetics model for data obtained under light and dark conditions, respectively.

In order to determine the mechanism involved in adsorption process the obtained kinetic data was also tested against the Weber-Morris intra-particle diffusion model [17]. Figure 1D presents the Weber-Morris plot as a functional dependence between the Ag^+^ ions adsorption and t^0.5^. Matching the experimental data to the model revealed the presence of three stages of sorption. The first one was the initial sharper step which can be assigned to external surface sorption, boundary layer diffusion effect and process of Ag^+^ ions reduction. The second linear step comes from gradual sorption with rate-limiting intra-particle diffusion mechanism. The last step can be related to sorption equilibrium. The possibility to measure the volume (thickness) of the external surface sorption is ensured by the y-axis intercept of the second sorption step line. Moreover, the value of the intra-particle diffusion coefficient determines the slope of this line. The characteristic of this plot allows to conclude that the Ag^+^ ions sorption process is mainly determined by silver adsorption on the external surface of casein. Gradual sorption of silver ions in the second stage indicates that the Ag^+^ ions diffuses and are absorbed into globule structure of the casein. Furthermore, the values of Gibbs free energy change (ΔG^0^) and distribution coefficient (K_d_) of the silver ions sorption by casein were calculated as −18.42 kJ/mol and 1826.00, respectively for process conducted in the presence of light as well as −16.98 kJ/mol and 1016.86 for dark conditions (Table 2). The negative value of Gibbs free energy indicates that the silver binding by casein is a spontaneous process. Pomastowski et al. [13] have studied the silver ion adsorption onto lactoferrin and the calculated value of ΔG^0^ was −16.06 kJ/mol, which is close to those one obtained in our study. However, K_d_ coefficient was calculated as 699,83, so we can assume that silver ions should more preferably adsorbed by caseins. This may be due to more complex caseins structure which form micelles in solutions while lactoferrin appears as a normal globular protein. 

**Figure 2:**
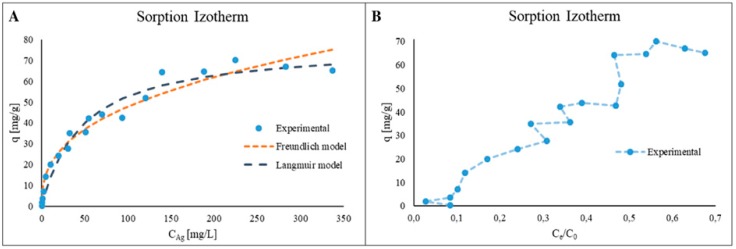
Isotherm of Ag^+^ ions sorption onto casein and fitting of the Freundlich and Langmuir isotherms models (**A**); sorption isotherm of Ag^+^ ions as a function of C_e_/C_0_ (**B**).

In order to provide a more detailed study of the mechanisms, which participate in the process of silver ions binding by casein, the adsorption isotherm study was performed. Figure 2A presents isotherm of the silver ions sorption process as a plot of sorption capacity change per Ag^+^ ions equilibrium concentration in solution, as well as the matching of experimentally obtained results to Freundlich and Langmuir models. Table 3 summarizes the calculated characteristic parameters for the used isotherm models. 

The calculated distribution coefficients (K_d_) were 8.62 L/g and 0.02 L/mg (or 8623 and 21000 as dimensionless values) for Freundlich and Langmuir models, respectively. It can be observed that the Langmuir model provides a better fit to the obtained experimental data. According to these results, it can be assumed that studied process have surface nature and a silver ions monolayer forms on the casein surface. However, isotherm as a function of C_e_/C_0_ (Figure 2B) [18] shows a more complex nature of the investigated process. Application of such dependence allows to identify mainly three dominant stages of silver ions sorption. At the first step formation of silver ions, monolayer occurs on the casein surface. Next, after applying initial concentration of silver ions of 10 mg/L a second layer formation starts by binding the silver ions to already adsorbed monolayer. Similarly, in the third step at initial Ag^+^ ions concentration of 200 mg/L a creation of third layer begins. The Freundlich and Langmuir models do not take into account the formation of multilayer or in our case the formation of silver nanoparticles. With such arrangement the interactions between the first layer of the silver ions and casein are the strongest and decreases with each successive layer. 

From isotherm study, the maximum sorption capacity for casein was calculated as 77.5 mg/g. Pabón et al. [19] perform an investigation of Zn^2+^ ions binding onto casein: the maximum sorption capacity was calculated as 32.00 mg/g for bovine casein at initial zinc ions concentration 500 mg/g. The same results for bovine casein sorption capacity towards Zn^2+^ ions was shown in the Pomastowski et al. [20] research (30.00 mg/g at initial zinc ions concentration 84.90 mg/L). As it was shown, casein has higher sorption capacity against Ag^+^ ions.

### 2.2. Spectroscopic Study of the Silver Binding Process 

**Figure 3:**
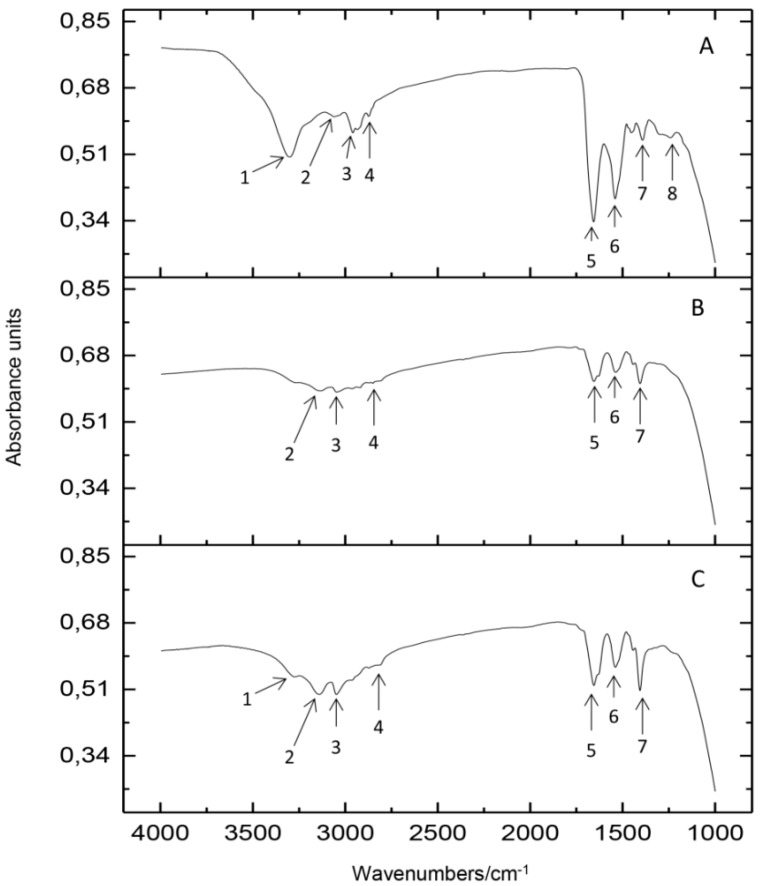
FT-IR spectra of casein before (**A**) and after silver binding reaction depending on the conditions of uptake—under dark (**B**) and light (**C**) conditions; Legend: (1) 3300–3400 cm^−1^, (2) 3150–3250 cm^1^, (3) 3050–3100 cm^−1^, (4) 2700–2800 cm^−1^, (5) 1600–1650 cm^−1^, (6) 1500–1550 cm^−1^, (7) 1400–1450 cm^−1^, (8) 1250 cm^−1^.

Spectroscopic study of native protein and Ag-CN complexes was applied to determine and describe the active chemical groups involved in the silver binding process. Figure 3 illustrates that registered FT-IR spectra for Ag-CN complexes are greatly different from that one for the native protein. Moreover, there are visible differences depending on the conditions of silver uptake process. In comparison with the spectra of casein before the binding process (Figure 3), the decrease in the intensity of all the spectral bands at υ = 1200–3400 cm^−1^ (1–8) range was observed. Based on the literature, the band around 1400–1450 cm^−1^ (7) might be assign to the N-O stretching vibrations as well as to the bending from the methyl (-CH_3_) groups and stretching vibrations from C-N groups [21,22]. The bands around 1500–1550 cm^−1^ (6) could originate from the deprotonated carboxyl (-COO^−^) groups and point out the presence of glutamic and aspartic acid in the casein structure. According to the [23,24] in casein structure as the dominant amino acid the glutamic acid was reported. Additionally, comparing the spectra before and after the silver binding process, the decreasing of band (6) intensity was observed which may indicate the crucial role of amino acid carboxyl groups in the described process. Furthermore, only in the sample of native protein, the band near the 1200 cm^−1^ was registered—disappearance of this band in samples after silver immobilization can be assigned to the participation of serine in the formation of Ag-CN complexes.

**Figure 4:**
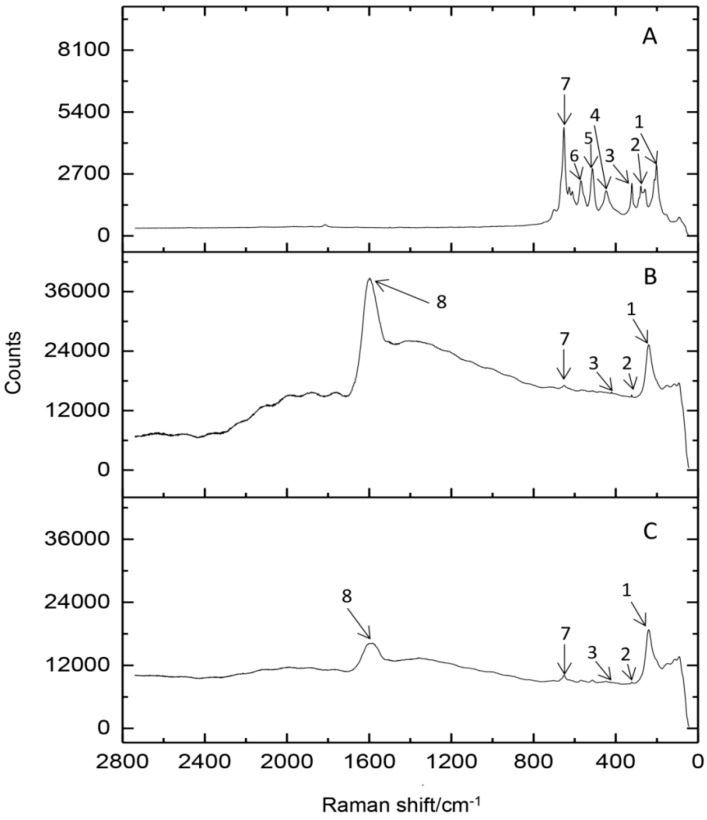
Raman spectra at the range 100–2800 cm^−1^ for native casein (**A**) and after the process of silver cations binding depending on the conditions of uptake –in the light (**B**) and in the dark (**C**); Legend: (1) 231 cm^−1^, (2) 253 cm^1^, (3) 302 cm^−1^, (4) 433 cm^−1^, (5) 489 cm^−1^, (6) 551 cm^−1^, (7) 648 cm^−1^, (8) 1589 cm^−1^.

Another approach applied to properly understand the Ag-CN binding process was Raman spectroscopy. In Figure 4, a Raman spectrum of casein and silver-casein complexes in broad range of 100–2800 cm^−1^ are shown. The spectral bands at 231 cm^−1^ (1), 253 cm^−1^ (2) and 301 cm^−1^ (3) visible in all samples can be assigned to the sulfur containing residues such as cysteine [25,26]. Bands no. (4–6) registered in the sample of native protein might be also related to the cysteine. The band at 648 cm^−1^ (7) is characteristic for the glutamic acid and its intensity is much higher after silver binding process—literature data [6,23] pointed out that α-casein contains 25 glutamic acid residues, which is dominant in the structure. Moreover, data from the FT-IR analysis also underline the crucial role of this amino acid in the Ag-CN complexes formation. Comparing the casein after silver immobilization (Figure 4B,C), the decrease in the intensity of some bands can be observed and it strongly depends on the conditions of Ag^+^ ions uptake process. The process occurring in the dark, resulted in the lower intensity of registered bands. Additionally, in the both samples after silver adsorption, the peak at 1589 cm^−1^ is present—it may derive from the -NH_3_^+^ group of lysine [25,27], which is also one of the most dominant amino acids in the structure of casein [6,23]. Taking into consideration previous scientific reports, Pomastowski et al. [13,20] have performed study of binding silver and zinc to lactoferrin and casein, respectively, we can assume the same binding mechanism in our study. Results from their spectroscopic study have indicated the pivotal role of carboxyl groups from aspartic and glutamic acid residues as well as phosphate groups in metal ions immobilization onto protein.

### 2.3. MALDI-TOF-MS Analysis

**Figure 5:**
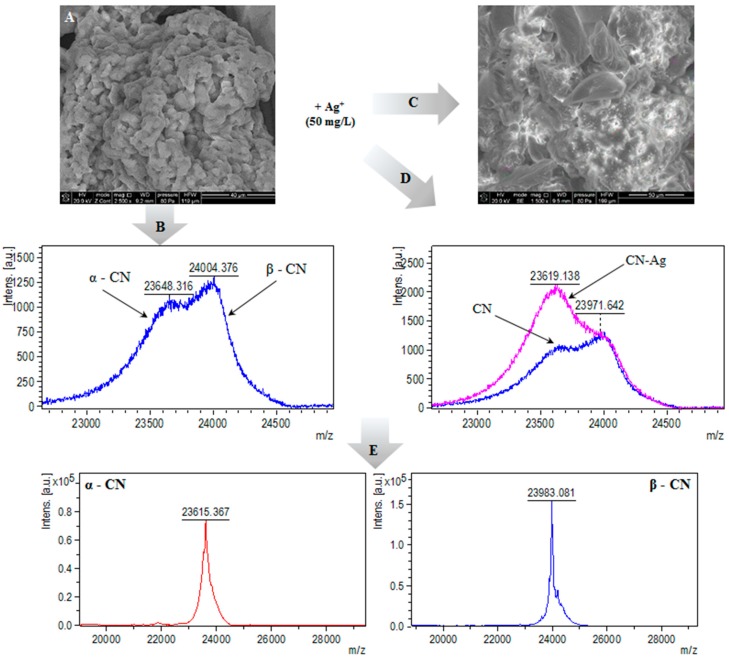
SEM image of native casein (**A**); mass spectrum of intact casein (**B**); SEM image of casein-silver complexes (**C**); mass spectrum of casein-silver complexes (**D**) and mass spectrum of intact α- and β-casein standard solutions (**E**), respectively.

In order to determine the casein masses before and after silver binding, the intact protein analysis using MALDI-TOF-MS (Figure 5B,D) in linear positive mode was performed. Average masses of intact casein was in the range of 23.648–24.004 kDa, which is in accordance with literature values [23]. Based on the MALDI-TOF-MS spectra, it can be noticed that α-casein and β-casein fractions were the dominant isoforms of casein in the investigated sample; no signal at *m/z*= 19 kDa (κ-casein) were observed. The Figure 5D shows the mass spectrograms of casein after the silver binding. After silver immobilization the signal at 23.6 kDa was promoted, which led to changes in signal distribution. Changes of the intensity of signals depending on the concentration of added silver show the dominant contribution of the alpha isomers in the described process. Pomastowski et al. [28] have carried out the separation of the bovine milk casein (α-, β- and κ-casein) components, and have used a MALDI-TOF-MS method for their detailed identification and characterization. The separation of the milk casein fractions was performed by HPLC gradient elution. After the chromatographic separation, the intact protein analysis for the obtained samples were applied. The received data indicate the molecular mass of α_s1_-, β-, and κ-casein as 23.610, 23.997, and 19.000 Da, respectively. Those values are strongly related to those obtained in present work. Recently, our research group [20] have also performed the study of binding zinc to casein and have applied the electrophoretic study to characterize the examined isoforms of protein. According to their data, and based on the peak intensity, it was noticed that α_s1_-casein and β-casein fractions were the dominant isoforms of casein.

### 2.4. Thermogravimetric Analysis

**Figure 6:**
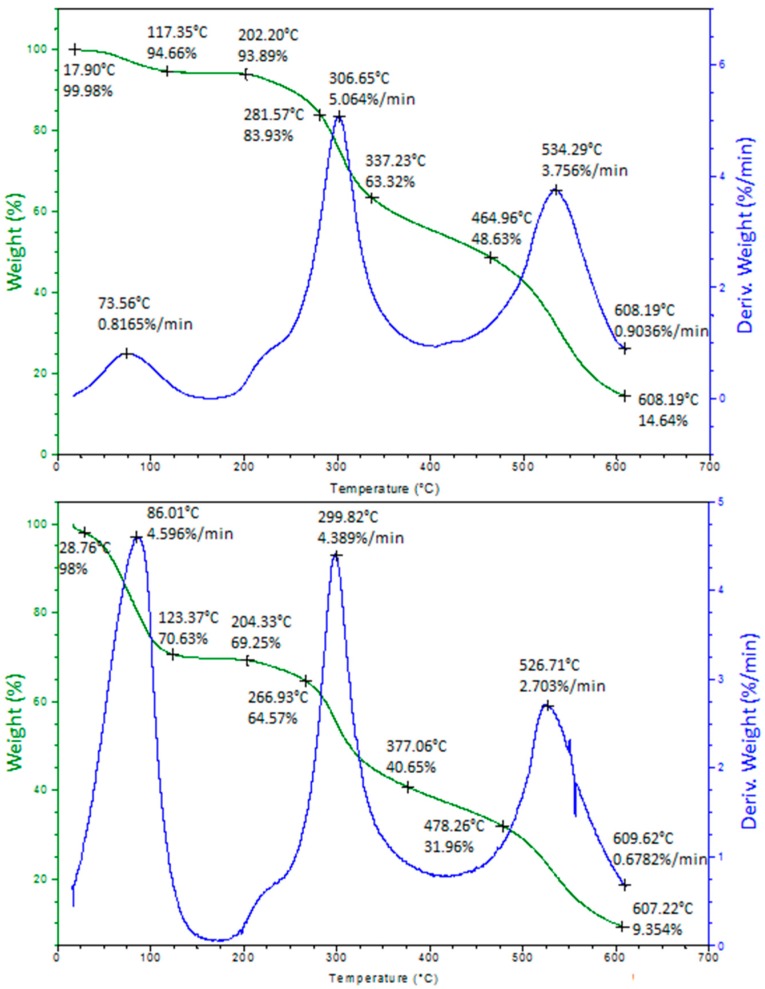
Thermogravimetry and derivative thermogravimetry analysis of silver-casein complexes (**up**) and native casein (**down**).

The stages of decomposition, temperature ranges, as well as the weight loss percentages of the sample are given in the Figure 6. The TG and DTG curves show that the decomposition of the native protein and complexes with silver proceeds in three main steps. The first stage is corresponding to the loss of lattice or coordinated water molecules—an initial loss of 30.75% and 6.11% is observed in the sample of casein and its complexes with silver, respectively. The weight loss rate for Ag-CN complexes is lower in comparison with the native protein (0.82%/min and 4.60%/min, respectively). Data from the first stage of thermal decomposition indicate that Ag-CN complexes are thermally more stable up to 202.2 °C than casein. The α-casein consist of the 214 amino acids residues, while the β-casein in its structure has 10 amino acids residues more. From all of them, the few amino acids are dominant: glutamic acid, proline, leucine, serine and lysine (α-casein) as well as proline, leucine, valine, glutamine and lysine (β-casein) [23,24]. It belongs to the group of phosphoproteins and forms residues of ortho- and pyrophosphate mainly at serine and threonine sites. 

Moreover, casein isolated from milk occurs mainly in the form of biocolloids formed from subunits consisting of individual fractions, linked together by a bridge comprising calcium ions, phosphates and citrates [20,23,29]. Therefore, the second stage of the process can be related with the thermal decomposition of protein structure and components such as amino acids. In the case of casein sample, there is an about 30% weight loss in the temperature range of 204.3–377.06 °C with the 4.39%/min rate. The same stage for Ag-CN occurs in the temperature range of 202.2–337.23 °C with a slightly higher rate (5.06%/min). It is known from the literature that thermal decomposition of amino acids cause the emission of mainly H_2_O and some NH_3_ [30,31]. Casein consists of glutamine and glutamic acid predominantly, and the dehydration of glutamic acid has been known for a long time as well as glutamine [31,32]. Already in 1932, Dunn and Brophy [33] have pointed the decomposition point of glutamic acid as a 247–249 °C. Recently, Weiss et al. have performed the study to investigate the thermal decomposition of a few amino acids [31]. According to their data, the one mol of H_2_O is the lost form the one mole of glutamic acid at 200°C temperature—the mass loss in the peak was observed as 12% [31]. In the case of glutamine, the precise 0.5 mol fractions of H_2_O and NH_3_ are decomposed from the amino acid structure at temperature 185 °C. The values obtained in our study are nearly close to the values from the literature. According to the [34], the optimum temperature for pyrolysis of casein was selected as 550 °C, in which the yield of pitch is higher. Data from our experiment indicate the pyrolysis of protein at 526.71 °C with the 2.70%/min rate. The pyrolysis of Ag-CN complexes were observed at 534.29 °C with a 3.76%/min. Moldoveanu et al. [35] have applied the TG-FTIR and TG-DTG-DTA approaches to analyze the thermal behavior of casein. Based on this paper [35], the casein is thermally stable between 0–172 °C which is closely related to values reached by our group. Moreover, the use of TG-FTIR method have allowed to describe the gaseous species eliminated from protein in the endothermic processes (CO_2_, H_2_O, NH_3_, HNCO and CO) as well as in the exothermal one (CO_2_ and H_2_O) [35].

### 2.5. SEM and TEM/EDX

**Figure 7:**
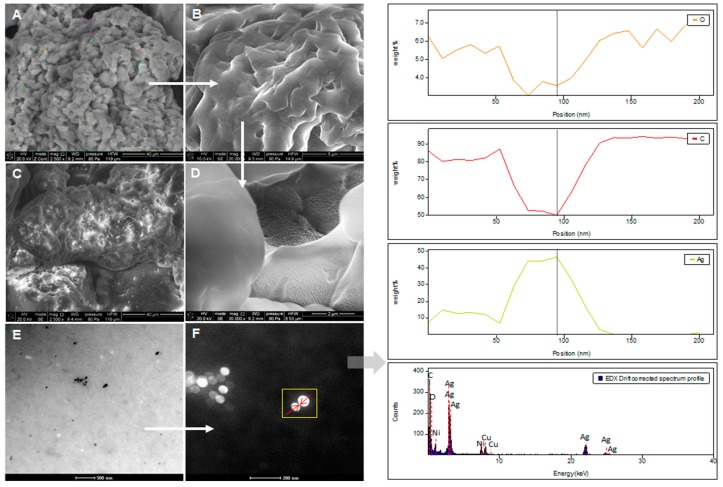
SEM image of native casein (**A**,**B**,**D**); SEM image of casein-silver complexes (**C**); TEM image of casein-silver complexes with the EDX spectra and EDS (Energy Dispersive Spectroscopy) elemental line scans (**E**,**F**).

SEM images of casein before and after the silver binding process are presented at the Figure 7A,B,D and Figure 7C, respectively. 

The TEM image of casein-silver complexes with the EDX spectra is shown at the Figure 7E,F and indicate the presence of silver(with approximately 3 keV, 22 and 25 keV signals) as a major element in the sample, which is in a good correlation with the literature values [36,37]. Beside Ag element, some amount of carbon, oxygen, copper and nickel elements were also detected, which can be result of the organic deposit present on the complexes surface as well as can be related to the sample preparation (Cu signals might correspond to the TEM grid). TEM analysis (Figure 7E,F) allows to observe the presence of many spherical in shape silver nanoparticles with size in the range of 4–100 nm.

### 2.6. Stability of Casein-silver Complex in Synthetic Physiological Fluids

The LOQ (Limit of Quantification) values for ICP-MS (Inductively Coupled Plasma Mass Spectrometry) analysis for Ag^+^ ions quantification was 1ppb. Measured concentrations of Ag^+^ ions after evaluation of Ag-CN complex in different synthetic physiological fluids were near the LOQ border therefore it was impossible to establish precise values (Table 4). Nevertheless, according to obtained results and value of previously calculated maximum sorption capacity q (17.00 ± 0.29 mg/g), a significant difference in concentrations of absorbed and released silver ions can be observed. Firstly, to make a conclusion about safety of the synthesized complex, it is worthy to mention that measured concentrations of free silver ions after incubation of complex in fluids for 24 h were significantly lower than estimated toxic levels of silver ions after oral exposure. Hadrup et al. summarized the levels of silver ions, subsequently leading to toxic effects on different organs, including lethal doses (LD_50_ for rabbits accounted for 800 mg/kg of silver/kg of bw/day, in rats the value was 280 mg/kg of silver/kg bw/day) [38]. Moreover, research of Greulich et al. [39] indicated that toxic effects of silver ions on human mesenchymal stem cells and peripheral blood mononuclear cells occurred in concentration range 0.5 to 5 ppm, which is higher in comparison with concentrations mentioned in Table 4. However, the formation of silver nanoparticle was confirmed and insignificant differences between absorbed and released ions could be probably explained by reduction of major part of ions to nanoparticles that can be a subject of further studies. In addition, studied complex can be stabilized probably due to re-binding of formed nanoparticles to casein, however surrounding them. As it was suggested previously, silver ions layers were formed on the surface of casein micelles. Then, reduction of absorbed ions to nanoparticles might occur with further formation of nanocluster and subsequently, being stabilized by casein probably due to interactions with free amine groups, imidazole ring or thiol groups. In addition, at pH close to pI of casein, micelles start to be more compact, thus preventing electrostatic repulsion between micelles [40], providing more efficient surface coverage of nanoparticles that was demonstrated in the study of gold nanoparticles stabilization by casein micelles by Liu et al. [41]. 

Secondly, data on casein digestibility in synthetic physiological fluids question the safety of the complex, as nanoparticles can be released upon casein digestion and aggregate (in pH 1.2) as was indicated by Pindáková et al. [42]. However, peptides, formed after casein digestion, can probably stabilize silver nanoparticles also due to interactions as in case of casein micelles. After all, casein digestion in synthetic gastric fluid (SGF) and synthetic intestinal fluid (SIF) was not studied in our work, thus leaving a gap for further research evaluation of stability of the complex.

## 3. Materials and Methods 

### 3.1. Kinetic Study of Silver Binding to Casein

Kinetic study of silver immobilization onto casein was performed by batch sorption method at room temperature (20 °C). Casein used in this study for the investigations was obtained from skim cow’s milk (Drzycim Dairy factory, Poland) using the method described by Pomastowski et al. [20]. Casein was suspended in 0.05 M ammonium bicarbonate solution to final concentration of 5 mg/mL and sonicated for 5 min. Next, 0.5 mL of casein suspension and 0.5 mL of 50 mg/L solution of AgNO_3_ was transferred to a 2 mL Eppendorf tube and was incubated for 2, 4, 6, 8, 10, 15, 20, 40, 60, 120, 720, 1440 and 2880 min. After the desired time has elapsed, the reaction was terminated by solutions centrifuging (RT, rpm, 8 min). Then, the supernatant was separated from the precipitate and the precipitate was washed by 0.5 mL of distilled water, again centrifuged and obtained supernatant was collected. Both supernatant fractions were combined. The silver concentration in obtained solutions was measured using Inductively Coupled Plasma Mass Spectrometer, ICP-MS 7500 CX (Agilent Technologies, Japan). The experiment was conducted in both light and dark conditions.

The amount of silver adsorbed by casein from solution was determined using the following equation:

q_t_ = (C_0_ − C) V/m (1)

where *q* is the amount of silver ions adsorbed at certain period of time (mg/g), *m* is the sorbent mass (g), *C*_0_ is the initial concentration of metal ions in aqueous solution (mg/L), *C* is the concentration of silver ions in aqueous solution at certain period of time (mg/L) and *V* is the volume of solution from which sorption occurs (L). 

The sorption effectiveness at certain period of time was calculated by the equation:

E% = 100*(C_0_ − C)/C_0_ (2)

where *E* is the sorption effectiveness (expressed in %).

#### 3.1.1. Modeling of Sorption Kinetic Data

In order to investigate the mechanism of metal ions sorption by casein, the zero, pseudo-first order kinetics and intra-particle diffusion models were used. 

The zero-order kinetics model was established by the equation:

C = C_0_ − k_0_ t (3)

where *C* is the concentration of silver ions in solution at time *t* (mg/L), *C*_0_ is the initial concentration of silver ions (mg/L), t is the time of adsorption duration (min), *k*_0_ is the rate constant ((mg/L)/min). The zero-order kinetic model was selected for description the linear segments separated on the kinetic curve.

The Lagergren pseudo-first order kinetic model [13,17] can be expressed by the following equations:

(4) $$q_{t}=q_{e}\left(1-e^{k_{1}t}\right)$$

where q_e_ is the amount of silver ions adsorbed at equilibrium (mg/g), q_t_ is the amount of silver ions adsorbed at time t (mg/g), k_1_ is the rate constant of pseudo-first order sorption kinetics (min^−1^), t is the duration of sorption (min).

The Weber-Morris intra-particle diffusion model [13,17] was used to determine the possible mechanism of sorption process, particularly in order to calculate the rate of inter-particle diffusion, according to the following equation:

q_t_ = A + K_ip_t^0.5^ (5)

where *q_t_* is the adsorbed amount at time t (mg/g), *A* is a constant that indicating the thickness of the boundary layer diffusion or external surface adsorption (mg/g), *K_ip_* is the diffusion rate constant ((mg/g)/t^0.5^).

#### 3.1.2. Determination of Thermodynamic Parameters

The distribution coefficient (*K_D_*) for the silver ions adsorption by casein was calculated based on the kinetic data at the equilibrium time according to equation:

K_D_ = q_e_/C_e_ (6)

where: *q_e_* is the number of ions adsorbed by protein at equilibrium time (mg/g), *C_e_* is the equilibrium concentration of silver in solution (mg/L). The distribution coefficient was used as an index of adsorbent affinity to metal ions sorption, because high *K_D_* value corresponds to higher sorption capacity of sorbent, and for calculation of the Gibbs free energy change (*∆G*^0^) [13,43]. The value of the Gibbs free energy change (*∆G*^0^) for the silver adsorption by casein was calculated according to the following relationship:

∆G^0^ = −RT lnK_D_ (7)

where *∆G*^0^ is the energy of adsorption in kJ/mol, *R* is the gas constant (8.314 J/mol·K), T is the adsorption absolute temperature in Kelvin (295 K) and K_D_ is the dimensionless distribution coefficient.

### 3.2. Isotherm Experiments

Aqueous solutions with increasing concentrations of silver ions (1, 5, 10, 20, 40, 60, 80, 100, 120, 140, 160, 180, 200, 250, 300, 350, 400, 450, 500 mg/L) were prepared. Then 0,5 mL of the respective silver solution was transferred to 2 mL Eppendorf tube and mixed with 0.5 mL of 5 mg/mL casein solution (in 0.05 M ammonium bicarbonate) through 24 h. After incubation the solutions were centrifuged (RT, rpm, 8 min). Then, the supernatant was separated from the precipitate and the precipitate was washed by 1 mL of distilled water, again centrifuged and obtained supernatant was collected. Both supernatant fractions were combined. The silver concentration in obtained solution was measured using Inductively Coupled Plasma Mass Spectrometer, ICP-MS 7500 CX (Agilent Technologies, Japan). 

#### Modeling of Sorption Isotherm Data 

In order to examine the mechanism of silver ions binding to the casein the several isotherm models have been applied for the experimental data: Freundlich isotherm, Langmuir isotherm and isotherm as function of *C_e_/C*_0_ [18], where *C_e_* is equilibrium concentration of the silver in the solution (mg/mL). The Freundlich isotherm model was established by the following equation:

(8) $$q=K_{F}C_{e}^{n}$$

where *K_F_* Freundlich rate constant (L/g) and *n* are empirical constants, that characterize the heterogeneity of the adsorption process. This model is generally used to describe the sorption process on the surface of heterogeneous and microporous adsorbents [18,44].

The Langmuir isotherm model can be established by the equation:

(9) $$q=\frac{q_{m}K_{L}C_{e}}{1+K_{L}C_{e}}$$

where *K_L_* is a Langmuir rate constant (L/mg) and *q_m_* is a maximum amount of silver that can be adsorbed in monolayer (mg/g). This model assumes that on the adsorbents surface a monolayer is create by molecules which interact with adsorption sites and do not interact with each other, there is no possibility to create a multilayer and the adsorption energy is constant [44].

Goodness-of-fit of the models to experimental kinetics and isotherm data was evaluated based on the correlation coefficient (R) and standard error (S) using the CurveExpert 1.37 software (Hyams Development, Huntsville, AL, USA). Accuracy of used models was determined based on the average relative approximation error (A_approx._).

In order to determine the most appropriate model for nonlinear equations, the Solver extension in Microsoft Excel (Microsoft Office 2007 Professional) was used. The value of error functions that was closest to unity for the coefficient of determination (R^2^) was the basis for model fitting. The calculations carried out by Solver is associated with fitting between the experimental data and the model equations according to [17]:

(10) $$R^{2}=1-\;\frac{\sum_{n-1}^{n}{\left(q_{e,exp}-\;q_{e,cal}\right)}^{2}}{\sum_{n-1}^{n}{\left(q_{e,exp}-\;q_{e,exp}\right)}^{2}}$$

where q_e,exp_ is experimental value of the absorbed silver ions at equilibrium and q_e,cal_ is the calculated amount of adsorbed silver from the model at equilibrium.

### 3.3. Physicochemical Characterization of Silver-Casein Nanocomplexes

#### 3.3.1. Fourier Transform Infrared Spectroscopy

Infrared spectroscopic study was carried out for verification of silver binding to casein. The infrared spectrum was measured in MIR range (FTIR Genesis II Mattson, Geneseo, NY, USA) using the thin layer method on CaF_2_ (Sigma-Aldrich, Poznan, Poland). Spectroscopic data was processed using WINFIRST software (Mattson, Geneseo, NY, USA).

#### 3.3.2. MALDI-TOF MS Analysis

The MALDI-TOF MS analyses were performed using chemicals at the highest commercially available purity supplied by Fluka Feinchemikalien (a subsidiary of Sigma-Aldrich, NeuUlm, Germany). Ground steel targets (Bruker Daltonik, Bremen, Germany) were used for sample deposition and the sinapinic acid was employed as matrix for MALDI analysis of intact proteins (dried droplet method) [28]. Protein Calibration Standards I and II (Bruker Daltoniks, Bremen, Germany) were used for external calibration. All the MS spectra were obtained using the MALDI-TOF/TOF mass spectrometer (Bruker Daltonik, Bremen, Germany) equipped with a modified neodymium-doped yttrium aluminum garnet (Nd:YAG) laser operating at the wavelength of 355 nm and frequency of 2 kHz. The system was controlled using the Bruker Daltonik software (flexControl and flexAnalysis). MS spectra of intact proteins were obtained in the linear positive mode in an *m/z* range of 15,000–30,000, applying an acceleration voltage of 25 kV. All mass spectra were acquired and processed using dedicated software flexControl and flexAnalysis, respectively (both from Bruker Daltonik).

#### 3.3.3. Thermogravimetric Analysis

Thermal analysis of casein and silver-casein complexes was carried out with the use of simultaneous TGA-DTA thermal analysis by TA Instruments type SDT 2960 (TA Instruments, Inc. New Castle, DE, USA). The samples were subjected to heating over a range of 0–600 °C with an air flow rate of 100 mL/min and heating rate of 10 °C/min.

#### 3.3.4. Raman Spectroscopy

Raman spectra were captured using a Raman Spectrometer with an optical microscope (Senterra, Bruker Optik, Ettlingen, Germany). Spectra were obtained at the range 100–2800 cm^−1^ and the wavelength at λ = 532 nm was used as excitation light, with the power of approximately 2 mW and the counting time spectrum at 30 s with 10 fM accumulation.

#### 3.3.5. Electron Microscopy (SEM, TEM) and Energy Dispersive X-Ray (EDX) Analysis

Distribution of silver-casein complexes size was investigated by transmission electron microscopy (FEI Tecnai F20 X-Twin, Hillsboro, OR, USA) and scanning electron microscopy (LEO 1430 VP) coupled with energy dispersive X-ray detector (XFlash 4010, Bruker AXS, Berlin, Germany). The samples for TEM analysis were dropped on the carbon-coated grid and the excess solution was removed, while for SEM analysis the powdered samples were used.

### 3.4. Application of Silver-Casein Complexes

#### Stability in Synthetics Physiological Fluids

For Ag-CN complex stability study, two types of synthetic physiological fluids were prepared: with and without enzymes, according to standards of Pharmacopeia by World Health Organization [45]. Silver-casein complex was synthesized by method, described in Section 2.1 with usage of 50 mg/L silver solution. Model gastric liquids were prepared by dissolving in 100-mL volumetric flasks 0.2 g of sodium chloride (POCH, Poland) without and with 0.32 g of pepsin (Sigma Aldrich, Poland) in 0.7 mL of concentrated hydrochloric acid (POCH, Poland) and filled up to the mark with distilled water from Milli-Q system (Millipore, USA). pH value for obtained fluids was about 1.2. Model intestinal fluids were prepared in 100-mL volumetric flasks by dissolving 0.68 g of potassium dihydrophosphate (POCH, Gliwice, Poland) in 25 mL of distilled water. Then 19 mL of 0.2 mol/L sodium hydroxide solution (POCH, Gliwice, Poland) without and with 1 g of pancreatine (Sigma Aldrich, Poznan, Poland) and 40 mL of distilled water were added. Solutions pH was brought up to 7.43 using 0.2 mol/L solution of NaOH and filled up to the mark with distilled water. For silver-casein complex stability experiment carrying out preliminary weighted silver-casein complex and 0.5 mL of selected synthetic liquid were placed to reverse spin tubes and transferred to shaker for 24 h. Then, mixtures were centrifuged for 10 min at 15,000 rpm. Supernatant from lower part of the tube was subjected to ICP-MS analysis for determination of free silver ions concentration. 

## 4. Conclusions

The study indicates that casein should uptake the silver ions from an aqueous solution with high effectiveness. Moreover, it was shown that presence of light promotes the silver elimination from solutions, which may be caused by photoreduction of the silver. Therefore, light conditions could be more effective for Ag-CN complex formation, as the more nanoparticles might form. Formation of silver nanoparticles with the size range 4–100 nm was confirmed by TEM images. 

Silver binding onto casein is a heterogeneous process with two main stages: initial rapid stage related to surface Ag^+^ ions adsorption onto casein and slower second stage of silver intraglobular diffusion and binding, which ends with equilibrium. Furthermore, the isotherm study indicates that silver adsorption onto casein has a complex nature, where multilayer of silver (silver nanoparticles) on casein surface forms.

FT-IR analysis indicates that carboxylic groups of aspartic and glutamic acid play a crucial role in silver binding onto casein. However, FT-IR and Raman spectra also show that functional groups of serine and cysteine might be the binding sites for silver ions. It should be noted that changes of the intensity of signals in MALDI-TOF MS spectra depending on the concentration of added silver show the dominant contribution of the α-casein isomers in the described process. The treatment of Ag-CN complex by synthetic physiological fluids does not provide to the release of a significant amount of free silver ions, which may be toxic. So, the synthesized complex could be used as a safe agent for food and agriculture purposes. However, further research should be done to investigate antibacterial properties against various types of bacteria strains, as well as possible toxicity of such preparations, which is our goal for future research.

## Figures and Tables

**Table 1:** Kinetic models parameters for the silver ions sorption by casein under light and dark conditions.

Zero Order Kinetics model		Pseudofirst-Order Kineticsmodel		Intra-Particle Diffusion Model	
***In light***					
*First step* k_0_ [mg L^−1^ min^−1^]	7.842	q_e_ [mg g^−1^] k_1_ [min^−1^] A_approx._[%]	17.009 0.166 13.286	A [mg g^−1^] K_ip_ [mg g^−1^min^−0.5^]	7.612 2.479
*Second step* k_0_ [mg L^−1^ min^−1^]	1.000				
***In dark***					
*First step* k_0_ [mg L^−1^ min^−1^]	7.040	q_e_ [mg g^−1^] k_1_ [min^−1^] A_approx._[%]	14.880 0.117 26.581	A [mg g^−1^] K_ip_ [mg g^−1^min^−0.5^]	8.692 1.538
*Second step* k_0_ [mg L^−1^ min^−1^]	0.692				

**Table 2:** The values of the distribution coefficient and the Gibbs free energy change of the metal ions sorption in light and dark conditions.

q_e_ [mg/g]	C_e_ [mg/L]	K_d_	T [K]	ΔG_0_ [kJmol^−1^]
*In light*				
17.01	9.31	1830.00	295	−18.42
*In dark*				
14.88	14.63	1016.86	295	−16.98

**Table 3:** Parameters of Freundlich and Langmuir approximation mathematical models of adsorption isotherms to the experimental data.

Freundlich Isotherm				Langmuir Isotherm			
K_F_ [L/g]	N	S	R^2^	K_L_ [L/mg]	q_m_ [mg/g]	S	R^2^
8.62	0.37	18.57	0.96	0.02	77.54	21.50	0.97

**Table 4:** Concentrations of Ag^+^ ions after evaluation of casein-silver (Ag-CN) complex in different synthetic physiological fluids measured by Inductively Coupled Plasma Mass Spectrometer (ICP-MS).

Type of Synthetic Fluid	[C] of Silver, ppb		
	1	2	3
SGF	1.526	0.781	1.358
SGF with pepsin	0.081	0.706	0.483
SIF	1.065	0.454	0.354
SIF with pancreatine	2.217	0.184	0.155

## Abbreviations

Ag-CN	Silver-casein
DTG	Derivative Thermogravimetry
EDS	Energy Dispersive Spectroscopy
EDX	Energy Dispersive X-ray detector
FT-IR	Fourier Transform Infrared Spectroscopy
HPLC	High Performance Liquid Chromatography
HSAB	Hard Soft Acid Base principle
ICP-MS	Inductevely Coupled Plasma Mass Spectrometry
MALDI-TOF MS	Matrix-Assisted Laser Desorption/Ionization Time Of Flight Mass Spectrometry
SEM	Scanning Electron Microscope
SGF	Synthetic Gastric Fluid
SIF	Synthetic Intestinal Fluid
TEM	Transmission Electron Microscopy
TGA	Thermogravimetric analysis
